# Distinct demographic profiles of spontaneous coronary artery dissection and coronary artery aneurysm: a single-centre experience from the United Arab Emirates

**DOI:** 10.3389/fcvm.2026.1845705

**Published:** 2026-07-10

**Authors:** Maria Khan, Yusra Jamil, Gohar Jamil, Adnan Agha

**Affiliations:** 1 Department of Medicine, Tawam Hospital, Al Ain, Abu Dhabi, United Arab Emirates; 2 College of Medicine, Dubai Medical University, Dubai, United Arab Emirates; 3 Cardiology Division, Department of Medicine, Tawam Hospital, Al Ain, United Arab Emirates; 4 Department of Internal Medicine, College of Medicine and Health Sciences, United Arab Emirates University, Al Ain, United Arab Emirates

**Keywords:** acute coronary syndrome, coronary artery aneurysm, pregnancy-associated, spontaneous coronary artery dissection, United Arab Emirates

## Abstract

**Background:**

Spontaneous coronary artery dissection (SCAD) and coronary artery aneurysm (CAA) are uncommon causes of acute coronary syndromes. Data from the Gulf Cooperation Council countries are limited. This study describes the clinical characteristics, management, and short-term outcomes of patients diagnosed with SCAD or CAA at a tertiary hospital in the United Arab Emirates.

**Methods:**

This retrospective observational study was conducted at Tawam Hospital, Al Ain, UAE (October 2018–May 2023). Cases were identified through dual ascertainment: systematic screening of electronic medical records using ICD-10 codes I25.41 (coronary artery aneurysm) and I25.42 (coronary artery dissection), supplemented by cross-referencing with the cardiac catheterization laboratory angiography registry. All identified records underwent independent chart adjudication by two investigators to confirm the diagnoses. Descriptive statistics were used throughout the study; no inferential comparisons were performed given the small sample size.

**Results:**

Of 109 records screened, 87 were excluded after chart adjudication (diagnostic misclassification), and 22 underwent a detailed review, yielding nine patients with confirmed diagnoses: six with CAA and three with SCAD. Patients with CAA were exclusively male, with a median age of 59 years (IQR 47.5–75.8), whereas all patients with SCAD were female, with a median age of 41 years (IQR 37.0–63.0). Two of the three patients met the criteria for pregnancy-associated SCAD (onset during pregnancy or within 12 weeks postpartum). One pregnancy-associated SCAD patient presented with cardiac arrest and required emergency coronary artery bypass grafting after a failed percutaneous intervention. The left anterior descending artery was involved in all assessable SCAD cases in this study. Conservative management was adopted in five of six (83.3%) patients with CAA and one of three patients with SCAD. One non-cardiovascular in-hospital death occurred in a patient with CAA admitted for palliative oncology care. All patients with documented follow-up reported improvement in symptoms.

**Conclusions:**

CAA and SCAD demonstrated distinct demographic and clinical profiles in this UAE cohort. The predominance of pregnancy-associated SCAD supports the need for heightened cardiovascular vigilance during the postpartum period. The absence of systematic screening for extracoronary arteriopathy represents a missed opportunity for diagnosis. These findings add to the limited Gulf regional data and support the development of prospective registries for uncommon coronary vascular disorders, including SCAD and CAA, in this population.

## 1 Introduction

Spontaneous coronary artery dissection (SCAD) is a non-atherosclerotic, non-traumatic, non-iatrogenic separation of the coronary arterial wall that creates a false lumen capable of compressing the true lumen and precipitating myocardial ischemia or infarction (1, 2). Once considered rare, SCAD is now recognized as a cause of 1%–4% of all acute coronary syndrome (ACS) presentations and up to 35% of myocardial infarctions in women younger than 60 years (2, 3). Large prospective registries have established that SCAD predominantly affects women (85%–91%) with a mean age of 45–54 years and a low burden of traditional cardiovascular risk factors (2–4). Fibromuscular dysplasia is identified in 43%–76% of patients when systematic screening is performed (4, 5).

Coronary artery aneurysm (CAA) is defined as the localized dilatation of a coronary artery segment exceeding 1.5 times the diameter of the adjacent normal segment (6, 7). The reported prevalence of CAA ranges from 0.3% to 5.3% in patients undergoing coronary angiography. CAA is etiologically heterogeneous: atherosclerosis accounts for the majority of adult cases, but Kawasaki disease sequelae, connective tissue disorders, and vasculitis are important non-atherosclerotic causes (6–8). This etiological diversity means that CAA cannot be classified as exclusively non-atherosclerotic, and its inclusion alongside SCAD in this study reflects a shared clinical presentation (ACS in the cardiac catheterization laboratory) rather than a shared pathophysiology.

Published data on SCAD from Gulf Cooperation Council (GCC) countries remain limited with the largest dataset being the multicenter Gulf SCAD (G-SCAD) Registry which spans across 30 centers in Saudi Arabia, the UAE, Kuwait, and Bahrain from 2011 to 2017, having 83 patients (9, 10). This registry documented a near-equal sex distribution (51% female), a high prevalence of pregnancy-associated SCAD among women (28.5%), and a higher rate of percutaneous coronary intervention (53%) compared with Western registries where conservative management predominates (9). Complementary data from individual case reports across the Gulf region have described pregnancy-associated SCAD and fibromuscular dysplasia-associated SCAD (11–13). Sub-analyses of the G-SCAD Registry have also examined psychosocial stressors including unemployment (14).

This study describes the clinical characteristics, angiographic findings, management strategies, and short-term outcomes of all the patients diagnosed with SCAD or CAA at single cardiac center in the UAE over a period of 4.5-years. The patients were identified through dual ascertainment by combining ICD-10-based screening of electronic medical records with cross-referencing against the cardiac catheterization laboratory angiography registry.

## 2 Methods

### 2.1 Study design and setting

This was a single-center retrospective observational study conducted at Tawam Hospital, Al Ain, Abu Dhabi, UAE. Tawam Hospital is a 468-bed government tertiary care facility affiliated with the United Arab Emirates University. It operates the only cardiac catheterization laboratory in the Eastern Region of Abu Dhabi Emirate and provides 24-hour primary percutaneous coronary intervention capability. The study was reported in accordance with the Strengthening the Reporting of Observational Studies in Epidemiology (STROBE) guidelines (15).

### 2.2 Ethical approval

The ethical approval was obtained from AlAin Region Human Research Ethics Committee with approval number MF2058-2025- 1170. The requirement for individual informed consent was waived by the ethics committee given the retrospective nature of the study, the use of de-identified data, and the minimal risk to participants.

### 2.3 Patient selection and case ascertainment

Cases were identified through dual ascertainment. First, all inpatient encounters at Tawam Hospital between October 2018 and May 2023 were screened using ICD-10 diagnostic codes I25.41 (coronary artery aneurysm) and I25.42 (coronary artery dissection). Second, the cardiac catheterization laboratory angiography registry was cross-referenced to identify cases that may have been coded under alternative diagnoses (e.g., acute coronary syndrome, acute myocardial infarction). These two strategies served complementary functions. Cross-referencing the angiography registry was intended to improve case-finding sensitivity by identifying cases that may have been coded under alternative diagnoses, whereas independent chart adjudication by two investigators improved diagnostic specificity by excluding miscoded records. ICD-10 screening flagged 109 records, of which 87 (79.8%) proved misclassified on adjudication, a specificity problem consistent with the known limitations of administrative coding for rare coronary conditions. The remaining 22 records underwent detailed clinical and angiographic review. Eight were confirmed as CAA or SCAD and were also present in the angiography registry. Cross-referencing the registry identified one further confirmed case (a 52-year-old man with CAA) that had not been captured by ICD-10 coding, yielding a final cohort of nine patients (six CAA, three SCAD). No case was identified by ICD-10 coding alone. Patients with iatrogenic coronary dissection, procedure-related coronary artery perforation, or miscoded diagnoses were excluded. Data were extracted from the hospital electronic medical record system using a structured case report form. Variables collected included demographic characteristics (age, sex, nationality, ethnicity, body mass index), presenting complaints (chest pain, dyspnea, cardiac arrest), admission vital signs (heart rate, blood pressure, respiratory rate, oxygen saturation), comorbidities (diabetes mellitus, hypertension, dyslipidemia, ischemic heart disease), laboratory investigations (troponin, D-dimer, complete blood count, renal function, thyroid function), electrocardiographic findings, coronary angiographic reports and conclusions, site and type of coronary lesion, risk factor assessment (emotional stress, intense exercise, Valsalva maneuver, estrogen/progesterone use, recent pregnancy, connective tissue disorder, fibromuscular dysplasia, inflammatory disorders, migraine, thyroid disorder, evidence of atherosclerosis), management strategy (conservative, percutaneous coronary intervention, coronary artery bypass grafting), discharge medications, inpatient complications, discharge outcome, and short-term follow-up data including timing of first outpatient review and 28-day readmission.

The high rate of ICD-10 misclassification (79.8%) and the unknown sensitivity of this screening strategy are acknowledged as limitations; genuine cases coded under alternative diagnoses may have been missed despite the supplementary angiography registry review.

### 2.4 Definitions

Coronary artery aneurysm was defined as localized dilatation of a coronary artery segment exceeding 1.5 times the reference vessel diameter on coronary angiography (6). Spontaneous coronary artery dissection was diagnosed based on angiographic demonstration of intimal flap, double lumen, or diffuse smooth narrowing consistent with intramural hematoma, in the absence of atherosclerotic plaque rupture or iatrogenic cause (1, 3). Pregnancy-associated SCAD was defined as SCAD occurring during pregnancy or within 12 weeks postpartum, consistent with the definition used in large SCAD registries (3, 16). Conservative management was defined as medical therapy without attempted revascularization during the index admission.

### 2.5 Statistical analysis

Nine patients. No denominator supports meaningful hypothesis testing. All analyses were therefore descriptive. Continuous variables were expressed as median (IQR) and mean ± SD. Categorical variables were expressed as frequency and percentage, with denominators specified where data were incomplete. Where risk factor assessment was not systematically performed or documented, the variable was reported as “not assessed” rather than absent, to avoid conflating missing data with negative findings. No inferential statistical tests, *p*-values, or confidence intervals were calculated, as the sample size precludes meaningful hypothesis testing. Data extraction and verification were performed using IBM SPSS Statistics (version 31.0; IBM Corp., Armonk, NY, USA).

## 3 Results

### 3.1 Study population

**Figure 1:**
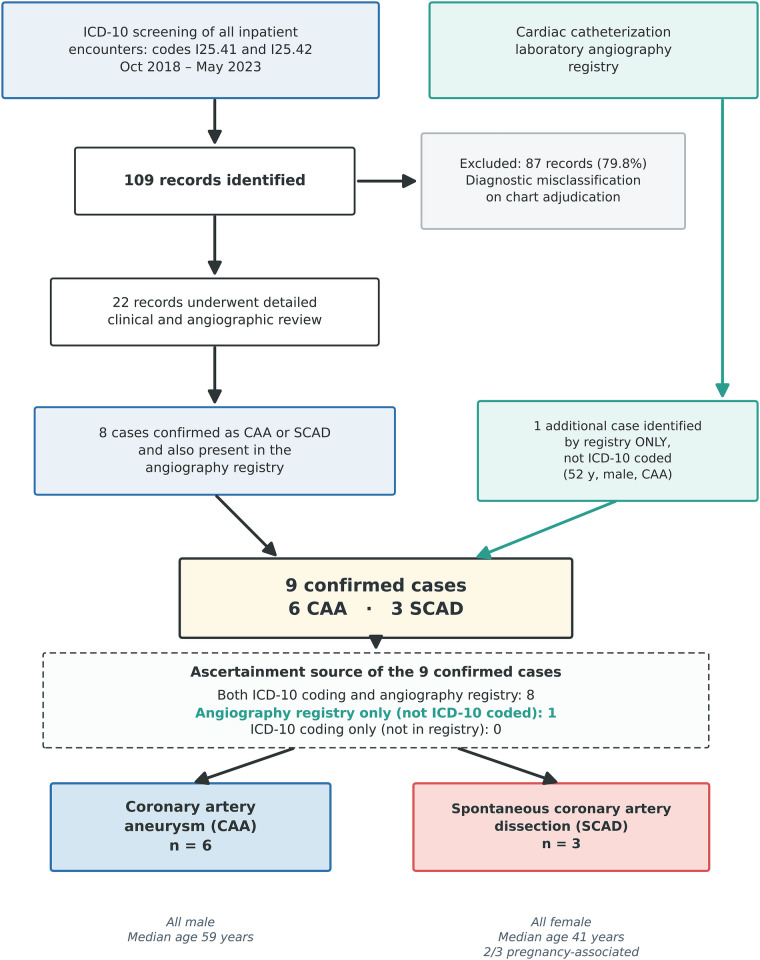
Patient ascertainment and selection flow diagram*.* Cases were identified by dual ascertainment at Tawam Hospital (October 2018 to May 2023): ICD-10 screening (codes I25.41 and I25.42) and independent cross-referencing of the cardiac catheterization laboratory angiography registry. ICD-10 screening identified 109 records, of which 87 (79.8%) were excluded for diagnostic misclassification on chart adjudication. Of the nine confirmed cases, eight were identified by both sources, one by the angiography registry alone (not captured by ICD-10 coding), and none by ICD-10 coding alone, yielding six CAA and three SCAD. CAA, coronary artery aneurysm; SCAD, spontaneous coronary artery dissection.

Systematic screening of inpatient encounters using ICD-10 codes I25.41 and I25.42, supplemented by angiography registry cross-referencing, identified 109 candidate records. After chart adjudication, 87 records (79.8%) were excluded due to diagnostic misclassification. Of the 22 remaining records, eight patients were confirmed as having CAA or SCAD, all of whom were also present in the angiography registry. Registry cross-referencing identified one additional confirmed CAA case not captured by ICD-10 coding, giving a final cohort of nine patients: six with CAA and three with SCAD (Figure 1).

### 3.2 Baseline characteristics

The baseline demographic and clinical characteristics are presented in Table 1. The median age of the overall cohort was 52.0 years (IQR 44.0–79.0). Six patients (66.7%) were male. Eight (88.9%) were Emirati nationals. All patients with documented ethnicity were of Arab origin (7/7, 100%). The six CAA patients were exclusively male with a median age of 59.0 years (IQR 47.5–75.8). The three SCAD patients were exclusively female with a median age of 41.0 years (IQR 37.0–63.0). Mean body mass index was 28.8 ± 7.5 kg/m^2^ in the CAA subgroup and 27.2 ± 2.2 kg/m^2^ in the SCAD subgroup.

**Table 1:** Baseline demographic and clinical characteristics.

Characteristic	Total (*n* = 9)	CAA (*n* = 6)	SCAD (*n* = 3)
Age, years, median (IQR)	52.0 (44.0–79.0)	59.0 (47.5–75.8)	41.0 (37.0–63.0)
Sex, male, *n* (%)	6 (66.7)	6 (100.0)	0 (0.0)
Sex, female, *n* (%)	3 (33.3)	0 (0.0)	3 (100.0)
Nationality, Emirati	8 (88.9)	5 (83.3)	3 (100.0)
BMI, kg/m^2^, mean ± SD	28.3 ± 6.3	28.8 ± 7.5	27.2 ± 2.2
Chest pain, n/N (%)	6/7 (85.7)	4/5 (80.0)	2/2 (100.0)
Dyspnea, n/N (%)	5/7 (71.4)	4/5 (80.0)	1/2 (50.0)
Cardiac arrest, *n* (%)	1 (11.1)	0 (0.0)	1 (33.3)
HR, bpm, median (IQR)	81.0 (74.5–105.0)	81.0 (68.0–98.0)	101.5 (91.2–111.8)
Comorbidities, n/N (%)			
Diabetes mellitus	0/9 (0.0)	0/6 (0.0)	0/3 (0.0)
Hypertension	1/6 (16.7)	1/5 (20.0)	0/1 (0.0)
Dyslipidemia	2/6 (33.3)	2/5 (40.0)	0/1 (0.0)

Continuous variables: median (IQR) or mean ± SD. Categorical variables: *n* (%) or n/N (%) with denominators specified where data are incomplete. No inferential tests performed.

Chest pain was the most common presenting complaint, documented in 6/7 patients with available data (85.7%). Dyspnea was recorded in 5/7 (71.4%). One SCAD patient presented with seizure and out-of-hospital cardiac arrest, with recorded vital signs of zero for systolic blood pressure and oxygen saturation at the time of emergency department arrival. No patient had documented diabetes mellitus. Hypertension was present in 1/6 assessable patients (16.7%), dyslipidemia in 2/6 (33.3%), and prior ischemic heart disease in 1/6 (16.7%); all within the CAA subgroup.

### 3.3 Risk factor profile

The risk factor profile is presented in Table 2. Documentation of precipitating factors was incomplete across the cohort. Emotional stress was assessed in four patients and documented as present in one (1/4). Pregnancy-associated status was the predominant identifiable precipitant in the SCAD subgroup: two of three SCAD patients (66.7%) met the study definition for pregnancy-associated SCAD (onset within 12 weeks postpartum). One was a 33-year-old Emirati woman who presented 14 days after caesarean delivery of twins with out-of-hospital cardiac arrest. The second was a 41-year-old Emirati woman who presented 10 days after caesarean delivery. Both events occurred within the first two weeks postpartum, well within the 12-week definition of pregnancy-associated SCAD. The third SCAD patient (85-year-old female) had no documented pregnancy association.

**Table 2:** Risk factors across the nine patients, with the number of patients assessed for each factor.

Risk factor/precipitant	Assessed (n/9)	Present, *n* (%) of assessed	CAA (*n* = 6)	SCAD (*n* = 3)
Emotional stress	4	1/4 (25.0)	1/4	0/0
Intense exercise	6	0/6 (0)	0/4	0/2
Valsalva/straining	7	0/7 (0)	0/5	0/2
Estrogen use	7	0/7 (0)	0/5	0/2
Pregnancy-associated	3	2/3 (66.7)	Not applicable	2/3
Connective tissue disorder	0	Not systematically screened	—	—
Fibromuscular dysplasia	0	Not systematically screened	—	—
Inflammatory disorder	0	Not systematically screened	—	—
Migraine	0	Not systematically screened	—	—
Thyroid disorder	0	Not systematically screened	—	—
Atherosclerosis (angiographic)	7	1/7 (14.3)	1/5	0/2

Values are the number of patients in whom the factor was present out of the number in whom it was assessed; percentages are calculated from assessed patients only. CAA, coronary artery aneurysm; SCAD, spontaneous coronary artery dissection.

Patients 6 and 9: both contributed no assessable risk-factor data (Patient 6 was admitted under palliative oncology care; Patient 9 had limited clinical documentation) and are counted as not assessed in every category.

Emotional stress: all four assessed patients were in the CAA group; no SCAD patient had this factor documented (0/0).

Pregnancy-associated: applies only to the three female SCAD patients; it was confirmed in two and not assessed in one (Patient 9), and is not applicable to the all-male CAA group.

Not systematically screened: connective tissue disorder, fibromuscular dysplasia, inflammatory disorder, migraine, and thyroid disorder were not systematically evaluated in any patient and are reported as not assessed rather than absent.

Fibromuscular dysplasia, connective tissue disorder, inflammatory disease, migraine, and thyroid disorder were not systematically screened for in any patient; these variables are therefore reported as “not assessed” rather than absent (Table 2). This represents a missed diagnostic opportunity, given that systematic screening identifies fibromuscular dysplasia in 43%–76% of SCAD patients in international registries (4, 5).

### 3.4 Coronary angiographic findings

Coronary angiography was performed in all nine patients. In the CAA subgroup, aneurysmal segments were identified in the right coronary artery (three patients), left anterior descending artery (two patients), and left circumflex artery (one patient). One patient had coronary artery fistulae with mid-LAD stenosis. One patient had a large RCA aneurysm (10 mm × 8 mm) with total occlusion distal to the aneurysm, presenting with ST-elevation myocardial infarction. Evidence of coronary angiographic atherosclerosis was documented in one of seven assessed patients (1/7), all within the CAA subgroup, underscoring the etiological heterogeneity of CAA in this cohort.

In the SCAD subgroup, two patients had confirmed angiographic findings. One demonstrated subtotal (99%) thrombotic occlusion of the proximal LAD with dissection extending to the first diagonal branch, consistent with Type 1 SCAD. The second had dissection of the distal LAD managed conservatively. The third SCAD patient (85F) had limited angiographic documentation available for detailed morphological classification; the diagnosis was based on the clinical presentation and available imaging, though diagnostic certainty is lower in the absence of intravascular imaging. All assessable SCAD cases involved the LAD territory.

### 3.5 Management and outcomes

Management strategies and outcomes are presented in Table 3. Conservative medical management was adopted in 5/6 CAA patients (83.3%); one was advised surgical intervention but declined. In the SCAD subgroup, one patient was managed conservatively, one underwent attempted PCI that was unsuccessful (wire entry into the false lumen) and was subsequently transferred for emergency coronary artery bypass grafting, and the third had limited treatment documentation.

**Table 3:** In-hospital management and short-term outcomes.

Characteristic	Total (*n* = 9)	CAA (*n* = 6)	SCAD (*n* = 3)
LOS, days, median (IQR)	2.88 (2.55–3.29)	3.02 (2.87–3.69)	1.69 (1.48–2.49)
Management			
Conservative	6 (66.7)	5 (83.3)	1 (33.3)
PCI attempted	1 (11.1)	0 (0.0)	1 (33.3)
Transfer for CABG	1 (11.1)	0 (0.0)	1 (33.3)
Complications, n/N (%)			
Any complication	2/7 (28.6)	1/5 (20.0)	1/2 (50.0)
Stroke	1	1	0
Cardiac arrest	1	0	1
Discharge outcome			
Discharged alive	8 (88.9)	5 (83.3)	3 (100.0)
Non-CV in-hospital death	1 (11.1)^a^	1 (16.7)^a^	0 (0.0)

a Non-cardiovascular death: 87-year-old male admitted under oncology with palliative care designation; death attributed to underlying malignancy, not CAA. LOS, length of stay; PCI, percutaneous coronary intervention; CABG, coronary artery bypass grafting; CV, cardiovascular, CAA, coronary artery aneurysm; SCAD, spontaneous coronary artery dissection.

Management categories are not mutually exclusive. The single SCAD patient who underwent attempted PCI was the same patient subsequently transferred for emergency CABG. Treatment documentation was incomplete for one SCAD patient, who is therefore not represented in the management rows. One CAA patient was advised surgical intervention but declined.

**Figure 2:**
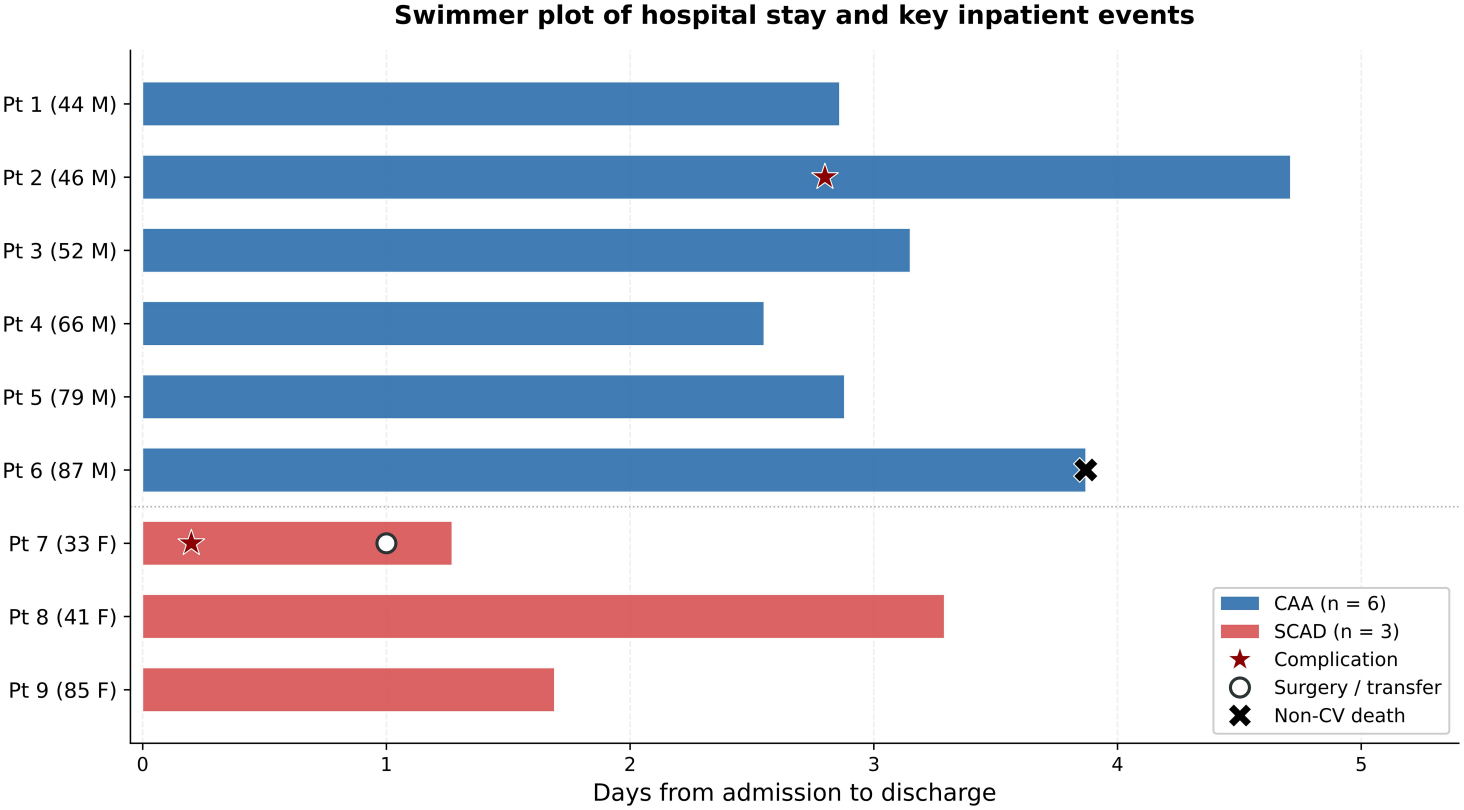
Swimmer plot of individual patient timelines. Each horizontal bar represents a single patient, color-coded by pathology (blue = CAA, red = SCAD). Bar length corresponds to length of hospital stay in days. Key clinical events (cardiac arrest, stroke, transfer for surgery, non-cardiovascular in-hospital death) are annotated with symbols. CAA, coronary artery aneurysm; SCAD, spontaneous coronary artery dissection.

Median length of stay was 3.02 days (IQR 2.87–3.69) for CAA and 1.69 days (IQR 1.48–2.49) for SCAD (Figure 2). Inpatient complications were documented in 2/7 patients with available data (28.6%): one CAA patient developed confusion, aphasia, and stroke with subsequent heart failure (ejection fraction 35%), and the pregnancy-associated SCAD patient experienced ventricular fibrillation and cardiac arrest.

One in-hospital death occurred. This was an 87-year-old male CAA patient who was admitted under oncology with a palliative care designation; the death was attributable to the underlying malignancy rather than to CAA, and is classified as a non-cardiovascular death. All surviving patients with documented follow-up reported symptom improvement at the first outpatient review.

**Figure 3:**
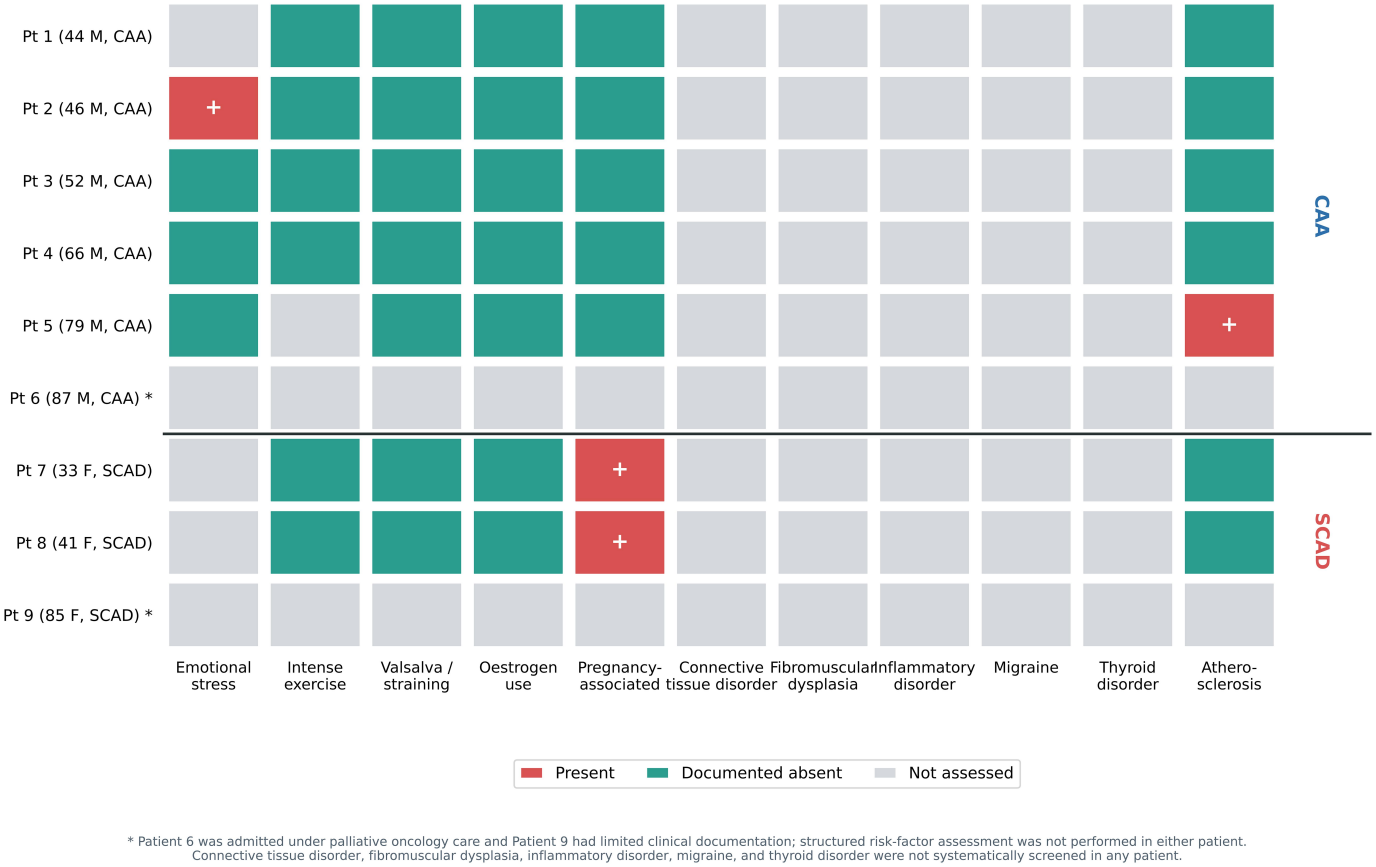
Patient-level risk factor heatmap. Patient-level risk factor and precipitant profile across the nine patients, grouped by pathology. Cells denote present, documented absent, or not assessed. Pregnancy-associated status is shown as a single category. Screening for extracoronary arteriopathy (fibromuscular dysplasia, connective tissue disorder, inflammatory disorder, migraine, and thyroid disorder) was not systematically performed in any patient and is therefore shown as not assessed. Pregnancy-associated SCAD was the predominant documented precipitant, present in two of three SCAD patients; angiographic atherosclerosis was documented in one CAA patient. CAA, coronary artery aneurysm; SCAD, spontaneous coronary artery dissection.

The patient-level distribution of documented risk factors and precipitants across the nine patients is summarized in Figure 3.

## 4 Discussion

This study describes nine patients with confirmed SCAD or CAA at a UAE tertiary hospital over 4.5 years. Three principal observations emerged: (1) CAA affected exclusively older males while SCAD affected exclusively younger females, consistent with established sex-specific patterns in the international literature (2–4, 6–8); (2) pregnancy-associated status was the predominant precipitant for SCAD, identified in two of three cases; and (3) conservative management was favored for CAA (83.3%), whereas SCAD management was individualized based on coronary anatomy and hemodynamic stability.

The sex dichotomy in this cohort mirrors international data. Large SCAD registries consistently report female predominance of 85%–91% (2–4). The G-SCAD Registry documented 51% female representation, a proportion attributed to differences in case ascertainment and potentially higher rates of diagnostic angiography in men with ACS in the Gulf region (9). The exclusive male representation in the CAA subgroup aligns with published male-to-female ratios of 2:1–4:1 in atherosclerosis-associated CAA (6–8).

The predominance of pregnancy-associated SCAD (two of three patients, 66.7%) exceeds the 4%–5% proportion in unselected SCAD registries, though it is comparable to the 28.5% reported in the G-SCAD Registry (9). This enrichment may reflect ascertainment bias inherent to a small sample. Both pregnancy-associated SCAD patients in this series presented acutely: one with cardiac arrest requiring emergency CABG after failed PCI. This severity profile is consistent with the observation that pregnancy-associated SCAD involves more proximal and multivessel disease, produces larger infarctions, and carries higher rates of cardiogenic shock compared with non-pregnancy-associated SCAD (16, 17).

Management patterns reflected guideline-concordant care. The conservative-first approach for CAA (83.3%) aligns with expert recommendations favoring antiplatelet therapy and surveillance imaging for asymptomatic or mildly symptomatic CAA (7, 8). For SCAD, current consensus recommends conservative management for clinically stable patients, acknowledging the high spontaneous healing rate and elevated technical failure rate of percutaneous intervention (1, 3, 18). The failed PCI in the pregnancy-associated SCAD case (wire entry into the false lumen) illustrates this procedural risk.

The absence of systematic screening for fibromuscular dysplasia and extracoronary arteriopathy in this cohort mirrors the G-SCAD Registry, which similarly reported no FMD in 83 patients (9). International registries identify FMD in 43%–76% of systematically screened SCAD patients (4, 5). The discrepancy likely reflects the absence of structured screening protocols in routine clinical practice in the Gulf region, rather than genuinely lower FMD prevalence, particularly given the case report from Oman demonstrating multivascular FMD underlying SCAD (11).

This study has important limitations. The sample size of nine patients precludes multivariable analysis, inferential comparisons, or generalizable prevalence estimates. Despite dual ascertainment combining ICD-10 screening with angiography registry cross-referencing, the sensitivity of the case identification strategy remains unknown: genuine cases coded under alternative diagnoses may have been missed. This is an inherent trade-off. Sensitivity was sacrificed for specificity. The 79.8% ICD-10 misclassification rate demonstrates poor specificity of administrative coding for these conditions, and future studies should consider supplementary catheterization laboratory log review. Several risk factor fields had high rates of missing data, with most precipitant and arteriopathy variables not systematically assessed. The third SCAD case (85F) had limited angiographic documentation, and in the absence of intravascular imaging, diagnostic certainty is lower. One in-hospital death occurred in a palliative oncology patient and was non-cardiovascular. This distinction matters. Attributing this death to CAA would misrepresent the disease-specific mortality of the condition. The single-center design limits generalizability beyond the catchment area of Tawam Hospital, and the short follow-up period does not capture long-term outcomes or recurrence, which is particularly relevant for SCAD where 5%–10% recurrence rates have been reported at 2 years (5).

CAA is etiologically heterogeneous, and one patient in this cohort had documented coronary atherosclerosis. The joint presentation of CAA and SCAD in this study reflects their shared mode of clinical detection at the cardiac catheterization laboratory rather than a unified non-atherosclerotic pathophysiology. This distinction should be maintained when interpreting the results.

These findings have three practical implications. First, clinicians managing peripartum chest pain in the UAE should maintain a low threshold for coronary angiography, even in women without traditional risk factors. Second, SCAD patients should undergo structured extracoronary vascular assessment, as endorsed by international guidelines (1, 3, 18). Third, the development of a UAE-wide prospective SCAD and CAA registry, building on the G-SCAD infrastructure, would enable robust epidemiological characterization in this underrepresented population.

## 5 Conclusion

This retrospective observational study identified nine patients with confirmed SCAD (*n* = 3) or CAA (*n* = 6) over 4.5 years at a UAE tertiary hospital. The conditions demonstrated distinct demographic profiles, with pregnancy-associated SCAD affecting younger women and CAA affecting older men; angiographic atherosclerosis was documented in one assessable CAA case. These findings are consistent with international registry data and contribute to the limited Gulf regional evidence base. Prospective multicenter studies with systematic arteriopathy screening and longer-term follow-up are needed.

## Data Availability

The raw data supporting the conclusions of this article will be made available by the authors, without undue reservation.
